# MonoGID: geometry and illumination aware enhancement with distillation for self-supervised monocular endoscopic depth estimation

**DOI:** 10.1186/s12880-026-02515-9

**Published:** 2026-06-29

**Authors:** Guodong Wei, Qi Bao, Tong Shi, Yuqin Li, Yu Miao, Wei He, Ying Tian, Zhengang Jiang

**Affiliations:** 1 Changchun University of Science and Technology, Changchun, Jilin China; 2 Jilin Provincial Key Laboratory of Intelligent Computing in Medical Image, Changchun University of Science and Technology, Changchun, Jilin China; 3 Zhongshan Institute of Changchun University of Science and Technology, Zhongshan, Guangdong China; 4 Jilin Agricultural University, Changchun, Jilin China

**Keywords:** Endoscopic monocular depth estimation, Self-supervised learning, Geometry-aware, Illumination-robust feature enhancement, Self-distillation

## Abstract

**Background:**

Monocular depth estimation in endoscopic scenes is a fundamental prerequisite for intraoperative 3D reconstruction and surgical navigation. However, factors such as weak textures, specular reflections, local overexposure, and dynamic illumination changes in endoscopic environments can undermine the effectiveness of photometric-consistency-based self-supervised learning methods, leading to insufficient structural representation and limited prediction accuracy.

**Methods:**

To address this issue, this paper presents a task-specific architectural extension of the EndoDAC framework for self-supervised monocular endoscopic depth estimation. Specifically, the proposed method adapts and integrates geometry- and illumination-aware feature enhancement, offline multi-generation self-distillation, and inference-stage structural fusion to improve feature representation, prediction refinement, and deployment efficiency under weak-texture and adverse illumination conditions. Experiments are conducted on the SCARED dataset for training and in-domain evaluation, while zero-shot cross-domain testing is performed on the Hamlyn dataset.

**Results:**

The results show that the proposed EndoDAC-based extension improves depth estimation performance on the SCARED dataset and achieves lower error-oriented metrics than the baseline on Hamlyn zero-shot evaluation, with threshold accuracy remaining comparable to the baseline.

**Conclusions:**

The proposed method demonstrates that task-specific adaptation of geometry- and illumination-aware feature enhancement, offline self-distillation, and inference-stage fusion can improve an EndoDAC-based self-supervised endoscopic depth estimation pipeline. These results support the effectiveness of the proposed architectural adaptation, while also indicating that further work is needed to improve cross-domain scale consistency and robustness under more challenging surgical conditions.

## Background

Monocular endoscopic depth estimation is important for intraoperative 3D reconstruction, surgical navigation, and augmented reality visualization in minimally invasive surgery [1, 2]. Compared with stereo endoscopes, monocular endoscopes are more compact and easier to deploy in narrow anatomical spaces, but recovering dense depth from monocular video remains ill-posed [3]. This problem becomes more challenging in endoscopic scenes because tissue surfaces often contain weak texture, non-Lambertian reflection, local overexposure, deformation, and instrument occlusion, which reduce the reliability of visual cues for depth recovery [4–7].

Deep learning methods have improved monocular depth estimation by learning dense representations from image data, but fully supervised approaches usually require accurate pixel-level depth annotations. Such annotations are difficult to obtain in real in vivo surgical environments because of sensor integration constraints, acquisition cost, and the lack of reliable ground-truth depth measurement [8]. Self-supervised learning therefore offers a practical alternative: it trains depth and pose networks from unlabeled videos by using view reconstruction and photometric consistency between adjacent frames [9, 10]. However, the same photometric assumption can be unreliable in endoscopic scenes, where illumination changes and non-Lambertian effects may introduce reconstruction errors that are not caused by incorrect geometry.

Existing monocular endoscopic depth estimation methods include traditional multi-view geometry, fully supervised learning, self-supervised learning, and more recently Transformer- or foundation-model-based approaches. Traditional methods such as ORB-SLAM provide geometric interpretability but often produce sparse or unstable reconstruction under weak texture and occlusion [11]. Fully supervised methods can predict dense depth maps, but their dependence on synthetic or ex vivo annotations limits generalization to real surgical videos [12]. Self-supervised methods reduce the need for depth labels, but early convolutional architectures remain limited in global structural modeling and boundary recovery [3, 13, 14]. Recent studies have introduced Vision Transformers, large-scale pretrained visual models, and contextual or adaptive depth estimation strategies to improve global representation, structural modeling, and transferability [15–17].

EndoDAC is a representative recent baseline in this direction. It adapts a visual foundation model to self-supervised endoscopic depth estimation through dynamic vector-based low-rank adaptation and additional convolutional modules, enabling joint depth and pose estimation without ground-truth depth labels or camera intrinsic parameters [18]. Despite its strong baseline performance, three aspects remain worth improving for practical endoscopic deployment. First, feature aggregation can be further adapted to preserve structural cues under weak texture and illumination disturbance. Second, the photometric training signal can still be noisy, which motivates additional prediction-level refinement. Third, parameter-efficient adaptation modules may introduce extra inference overhead, making deployment-stage structural fusion useful [19, 20].

Based on the above analysis, this paper presents an EndoDAC-based method for self-supervised monocular endoscopic depth estimation. The method focuses on endoscopy-oriented architectural adaptation by integrating geometry- and illumination-aware feature enhancement, offline multi-generation self-distillation, and inference-stage structural fusion into a unified self-supervised pipeline. The main contributions are summarized as follows:

1. We integrate a geometry- and illumination-aware feature enhancement structure into the EndoDAC depth estimation pipeline. Specifically, GA-BiFPN injects coarse depth-derived structural cues into BiFPN-based multi-scale feature fusion through a gated mechanism, aiming to strengthen structural consistency in weak-texture and visually ambiguous endoscopic regions. ISA is introduced after the Conv Neck to reorganize bottleneck features under endoscopic lighting disturbances by combining illumination-aware channel recalibration with lightweight semantic feature extraction. This combined design improves feature robustness under weak texture, specular reflection, local overexposure, and dynamic illumination changes.
2. We adapt offline multi-generation self-distillation to the proposed EndoDAC-based self-supervised endoscopic depth estimation pipeline. The teacher-student refinement is applied to depth predictions across generations, providing additional soft supervision without requiring ground-truth depth annotations. This strategy is used to improve prediction consistency and reduce large local errors caused by unreliable photometric supervision in challenging endoscopic regions.
3. We apply an inference-stage structural fusion acceleration scheme to reduce deployment overhead. Specifically, LoRA weight fusion and Conv–BN fusion are performed after training to fold additional branches and cascaded operators into equivalent compact forms for inference. This component is used as an engineering optimization for runtime efficiency rather than as a new depth estimation formulation.

## Methods

### Overall architecture

In self-supervised monocular depth estimation, geometry-reprojection-based learning frameworks have become the mainstream paradigm [21]. The core idea is to construct supervisory signals from view reconstruction relationships between adjacent video frames, thereby enabling end-to-end optimization without requiring ground-truth depth labels. However, when such methods are applied to endoscopic videos, they still face several prominent challenges. On the one hand, non-Lambertian phenomena such as specular reflections, shadows, and local overexposure are common in endoscopic imaging. These factors violate the assumption of photometric consistency across views and interfere with the geometric constraints imposed by reprojection error [22]. On the other hand, in large weak-texture or smooth soft-tissue regions, the effective gradient information available for reconstruction is limited, which makes the model rely more heavily on feature representation capability and structural priors [23]. In addition, deployment scenarios for real-time navigation and intraoperative applications impose stricter requirements on inference latency. Therefore, how to further improve inference efficiency while maintaining prediction accuracy is also an important issue that such methods need to address.

To address the above challenges, EndoDAC introduces visual foundation model priors into a self-supervised monocular depth estimation framework and generally follows a closed-loop training pipeline of “depth prediction–pose and intrinsics estimation–geometric reprojection reconstruction” [18]. Specifically, the DepthNet predicts multi-scale depth maps from the target frame, while the Pose–Intrinsics Net estimates the relative pose and camera intrinsics from adjacent frames. Based on the estimated geometric relationships, the source frames are reprojected to the target viewpoint to generate reconstructed images, and the network is trained end-to-end by minimizing the reconstruction error. Compared with traditional convolutional network-based methods, EndoDAC demonstrates advantages in complex scene structure modeling and global representation capability, providing an effective baseline framework for self-supervised endoscopic depth estimation.

However, when this baseline is applied to endoscopic scenes, it still exhibits three major limitations. First, when dealing with weak-texture tissue regions and adverse illumination disturbances such as specular reflections and overexposure, the feature representation capability of the existing framework remains limited, making it difficult to fully balance local structural detail preservation with robust modeling under complex imaging conditions. Second, the self-supervised optimization process based on photometric reprojection constraints remains susceptible to noisy supervisory signals, thereby limiting further improvement in depth prediction accuracy. Finally, the parameter-efficient fine-tuning structure in the baseline still introduces additional computational overhead during inference, which is unfavorable for deployment in scenarios with stringent real-time requirements [24].

**Fig. 1:**
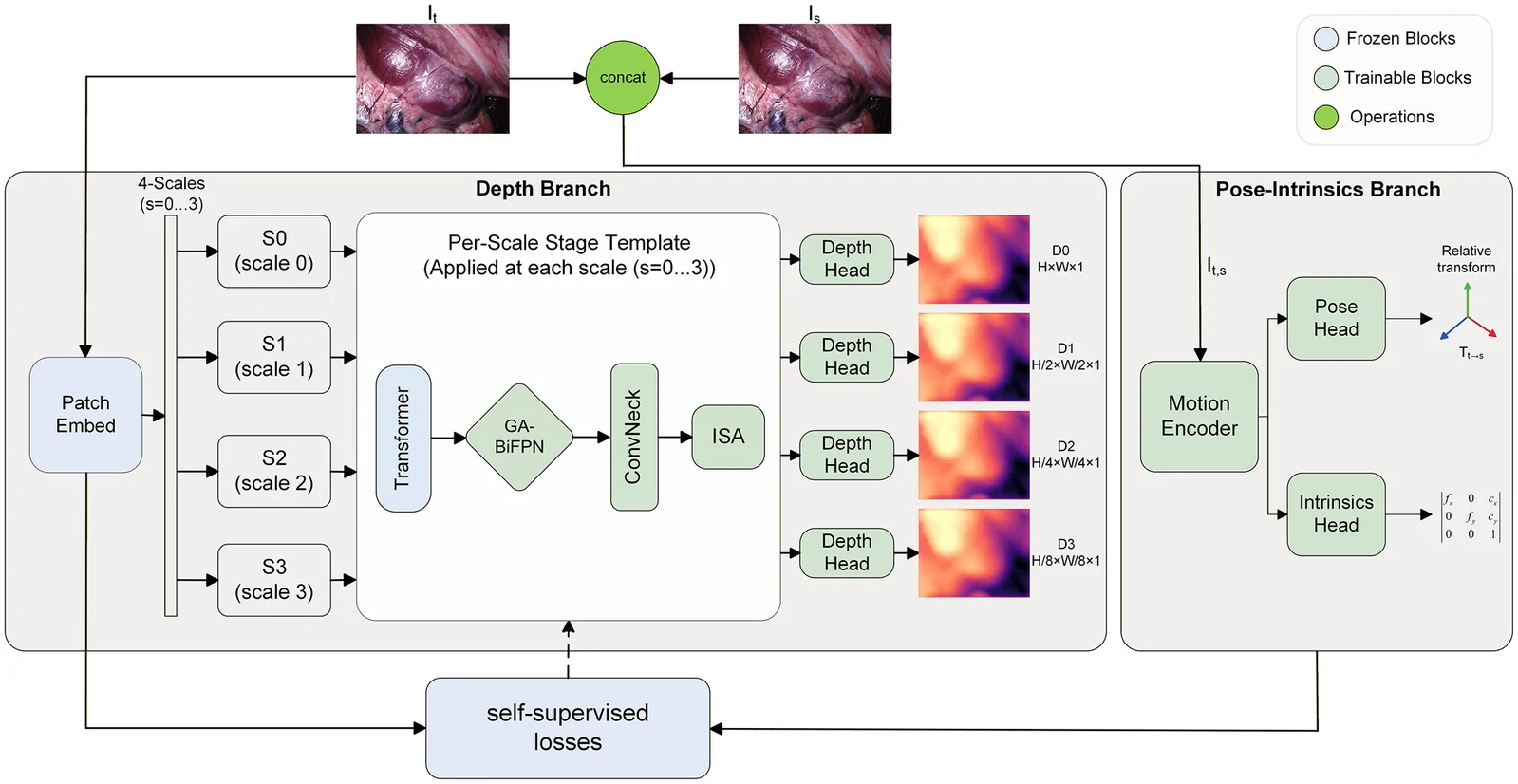
Overall architecture of the proposed model

Building upon the EndoDAC baseline [18], the proposed method preserves the original self-supervised photometric reprojection formulation and improves the pipeline mainly at the levels of feature representation, training refinement, and inference deployment. The overall network architecture is shown in Fig. 1. In terms of feature representation, GA-BiFPN and ISA are integrated into DepthNet to improve multi-scale structural fusion and illumination-robust bottleneck enhancement under weak-texture and adverse illumination conditions. In terms of training refinement, offline multi-generation self-distillation is adopted to provide additional soft depth supervision across generations. During deployment, structural fusion is applied only at the inference stage to reduce the overhead introduced by additional branches and cascaded operators.

### Geometry-aware and illumination-robust feature enhancement module

To enhance the robustness of the EndoDAC baseline under endoscopic illumination variations and weak-texture conditions, we integrate two complementary feature enhancement modules into the DepthNet backbone: GA-BiFPN (Geometry-Aware BiFPN) and ISA (Illumination-Semantic Attention). GA-BiFPN is inserted between the Transformer encoder and the Conv Neck, where coarse depth-derived geometric cues are injected during multi-scale feature fusion. ISA is placed after the Conv Neck and before the Decoder, where illumination-aware channel recalibration and semantic feature enhancement are jointly used to provide more stable bottleneck features for depth decoding.

#### Geometry-aware BiFPN (GA-BiFPN)

We use BiFPN [25] from the EfficientDet family as the multi-scale aggregation backbone and add a task-specific geometric injection branch for self-supervised endoscopic depth estimation. This design makes the multi-scale fusion process more sensitive to depth-related structural cues in weak-texture endoscopic regions. Let the multi-scale features extracted from the Transformer be denoted as $\{P_i^{in}\}$, with the resolution progressively decreasing from lower to higher levels. GA-BiFPN iteratively performs bidirectional fusion through top-down and bottom-up pathways, enabling thorough integration of high-level semantic information with low-level spatial details. To avoid imbalance in the contributions of different scales caused by simple summation, we assign each input $\{I_j \}$ at a fusion node a learnable weight $w_j$, and apply non-negative normalization to obtain a stable weighted fusion result:

1 $$\hat{w}_j = \frac{\phi(w_j)}{\varepsilon + \sum_k \phi(w_k)},\quad O = \sum_j \hat{w}_j I_j$$

where $\phi(\cdot)$ denotes the ReLU-based non-negative activation, and *ɛ* is a small constant.

On this basis, we further introduce a geometric branch to explicitly provide structural priors: a coarse depth map $\tilde{D}$ is predicted from the deepest feature $P_5$, which contains the strongest semantic representation, through a lightweight depth head:

2 $$\tilde{D} = f_d(P_5), \quad f_d: \mathrm{Conv}_{1\times 1} \to \mathrm{BN} \to \mathrm{ReLU} \to \mathrm{Conv}_{1\times 1}$$

Subsequently, geometric cues are computed from $\tilde{D}$, for which the depth gradient magnitude [26] is adopted in this paper:

3 $$G = \sqrt{(\partial_x \tilde{D})^2 + (\partial_y \tilde{D})^2}$$

**Fig. 2:**
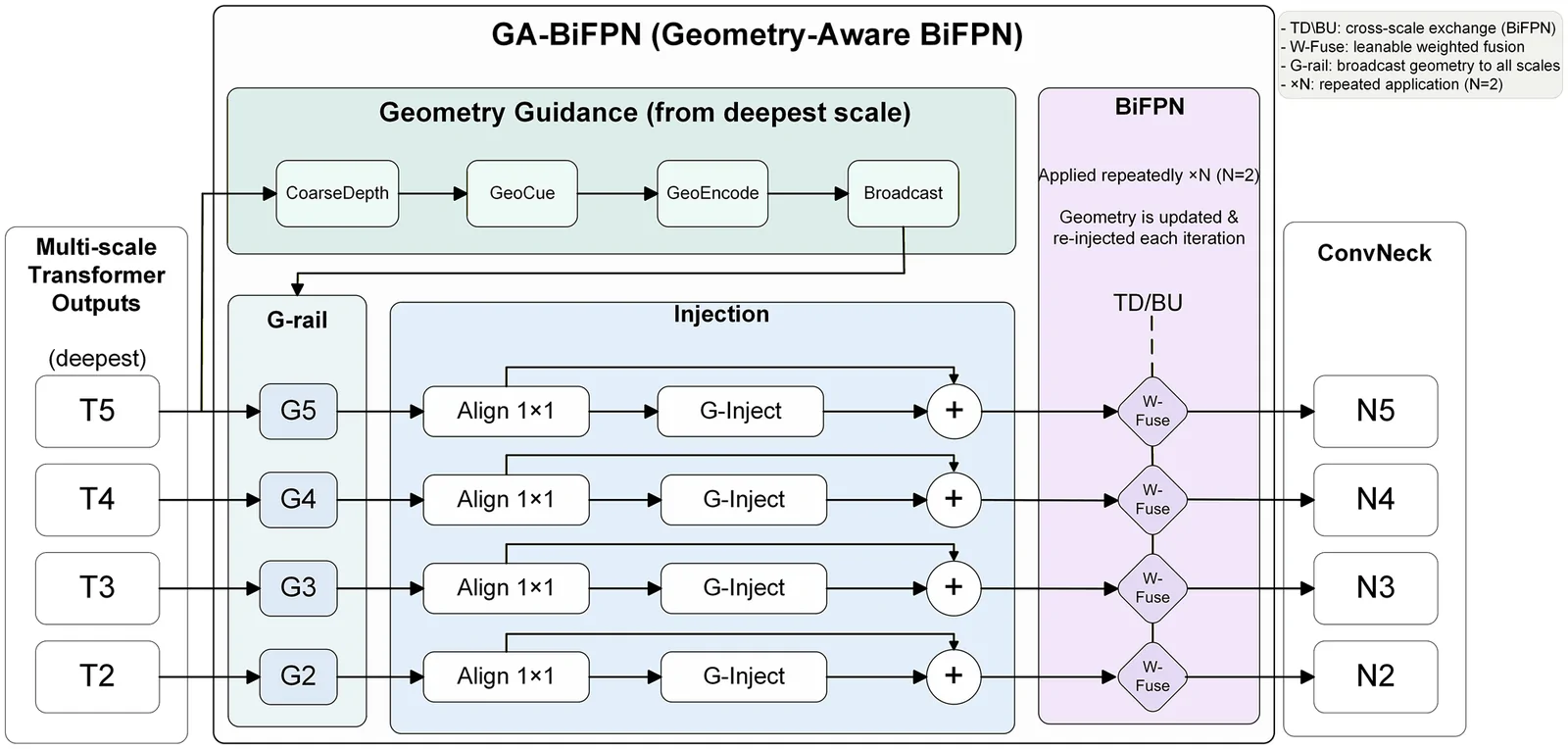
Architecture of the GA-BiFPN module

The geometric cue *G* is mapped by the geometric encoder $f_g(\cdot)$ to the same channel dimension as the BiFPN features, and is further aligned across scales to obtain $F_{geo}^{(i)}$. After each fusion output $F_{orig}^{(i)}$, a gated injection operation is performed:

4 $$\begin{aligned}&M^{(i)} = \sigma\left(\mathrm{Gate}\left(F_{geo}^{(i)}\right)\right),\cr &\quad F^{(i)} = F_{orig}^{(i)} \odot M^{(i)} + F_{geo}^{(i)} \odot \left(1 - M^{(i)}\right)\end{aligned}$$

where $\mathrm{Gate}(\cdot)$ denotes a lightweight *1 × 1* mapping, and *σ* denotes the Sigmoid function. As shown in Fig. 2, GA-BiFPN introduces a geometric branch on top of the bidirectional multi-scale fusion of BiFPN, and injects geometric cues through a gating mechanism after the fusion output at each scale.

This injection strategy allows the network to adaptively regulate the contribution of structural information according to the reliability of geometric cues, strengthening structural constraints in regions with clear geometric boundaries while preserving the original fused features in geometrically ambiguous areas, thereby improving the stability of geometric learning under weak-texture and unstable-illumination conditions. The resulting multi-scale features are then passed to the baseline Conv Neck for subsequent high-frequency enhancement.

#### Illumination-semantic attention (ISA)

To mitigate the influence of specular reflections, shadows, and local overexposure on feature representations in endoscopic images, we add the ISA module between the Conv Neck and the decoder to reorganize bottleneck features under endoscopic illumination disturbances. ISA follows the general idea of attention-based feature recalibration, but adapts it to the endoscopic setting by combining illumination-aware channel weighting with lightweight semantic feature extraction. Let the input feature be denoted as $x \in \mathbb{R}^{H \times W \times C}$. ISA adopts a parallel dual-branch structure: the illumination branch models the global illumination distribution and performs channel recalibration, while the semantic branch extracts high-level semantic patterns related to tissue structures. The outputs of the two branches are fused to produce an enhanced feature *y* in a residual manner.

The illumination branch is built upon SE attention [27]. First, a channel descriptor is obtained through global average pooling:

5 $$z_c = \frac{1}{HW} \sum_{h,w} x_{h,w,c}$$

The channel weights are then obtained through two fully connected layers and activation functions:

6 $$s = \sigma\left(W_2 \delta\left(W_1 z\right)\right)$$

The input is then channel-wise reweighted to obtain $x^{illum} = x \odot s$. This process adaptively suppresses abnormal channel responses caused by illumination-induced artifacts by leveraging global contextual information.

The semantic branch is implemented as a lightweight feature extractor. Inspired by the efficient design of MobileNetV3 [28], it is centered on depthwise separable convolution [29] (implemented as *1 × 1*Conv $\rightarrow 3 \times 3$ Depthwise Conv $\to 1 \times 1$ Pointwise Conv), and uses a convolutional head (*3 × 3* Conv + *1 × 1* Conv) at the end to generate the semantic-enhanced feature $x^{sem} = f_{sem}(x)$. This branch is intended to strengthen the representation of structural patterns such as blood vessels and mucosa, thereby compensating for the possible attenuation of structural information caused by illumination suppression alone.    

Finally, ISA concatenates the outputs of the two branches along the channel dimension and applies a *1 × 1* convolution to complete channel fusion and restoration:

7 $$u = \mathrm{Conv}_{1\times 1}\left([x^{illum}; x^{sem}]\right)$$

and adopts a residual connection and normalization to stabilize training:

8 $$y = \mathrm{BN}(u + x)$$

**Fig. 3:**
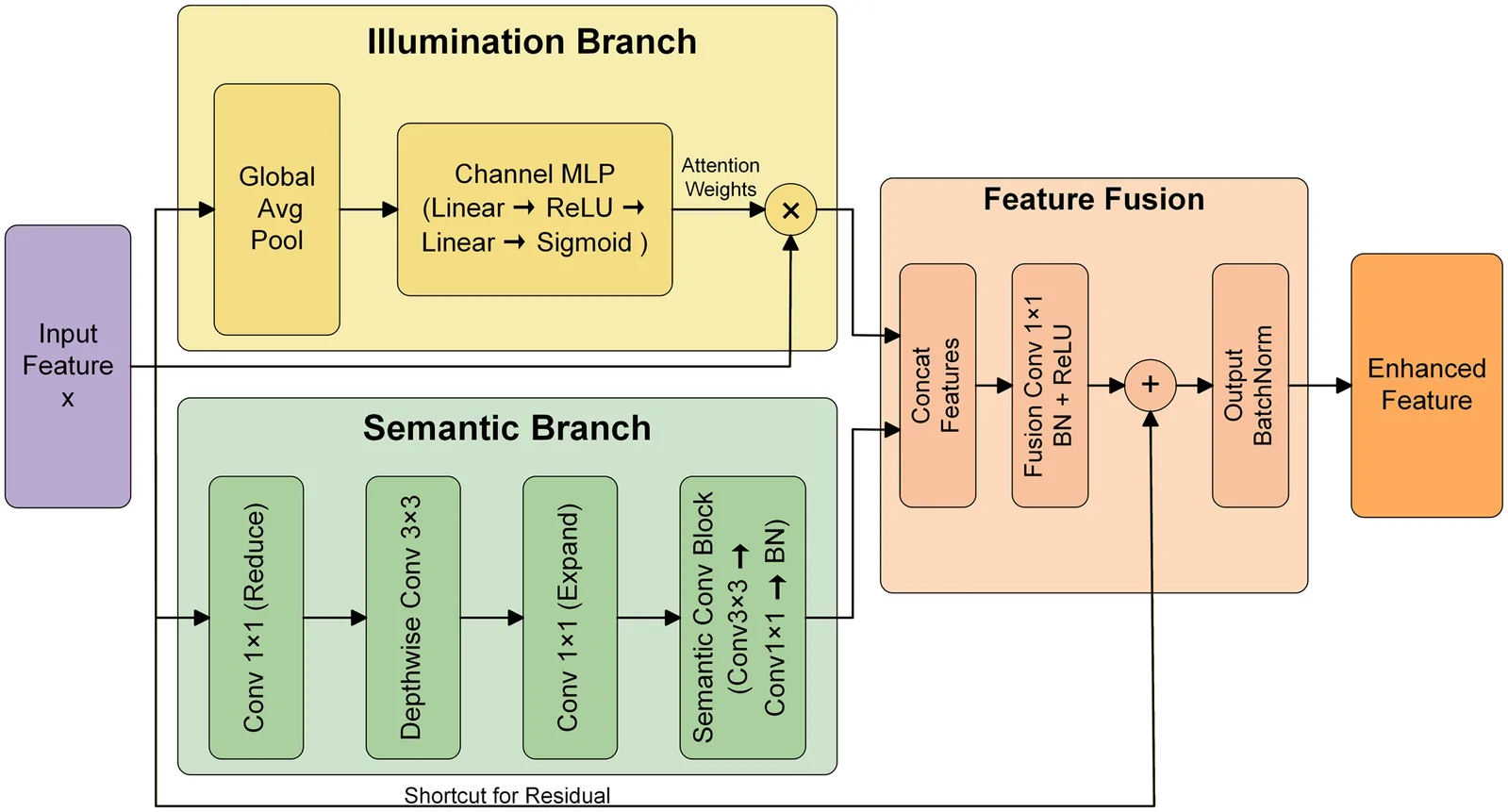
Architecture of the ISA module

As shown in Fig. 3, the ISA module adopts a parallel structure with an illumination branch and a semantic branch to jointly capture illumination and semantic cues. The illumination branch performs channel recalibration through the SE attention mechanism, while the semantic branch extracts high-level semantic features through depthwise separable convolutions. After fusion, the enhanced feature is provided through a residual connection.

The residual mechanism enables ISA to incrementally enhance features while preserving the original information flow. Batch Normalization (BN) further stabilizes the output distribution, providing the Decoder with more consistent inputs under large illumination variations.

#### Motivation of the feature enhancement design

The feature enhancement design is motivated by the failure modes of photometric self-supervision in endoscopic scenes. In view-synthesis-based self-supervised depth estimation, the target frame is reconstructed from adjacent frames according to the predicted depth, relative pose, and camera intrinsics. The photometric discrepancy between the target image and the reconstructed image then provides the main supervisory signal for depth learning:

9 $$\mathcal{L}_{photo} = \sum_{p} \rho\left(I_t(p)-\hat{I}_{s\rightarrow t}(p)\right)$$

where $I_t$ denotes the target image, $\hat{I}_{s\rightarrow t}$ denotes the source image reconstructed into the target view, *p* denotes a pixel location, and $\rho(\cdot)$ denotes the robust photometric penalty used in the self-supervised objective.

However, in endoscopic images, this supervisory signal can become unreliable in two typical situations. First, weak-texture or homogeneous tissue regions provide limited image gradients, making it difficult for photometric reconstruction to impose stable local geometric constraints. Second, specular reflection, local overexposure, shadow, and illumination variation may produce photometric residuals that are not caused by geometric misalignment, thereby introducing noisy supervision for depth optimization.

Based on this observation, GA-BiFPN and ISA are designed to improve feature representation from two complementary aspects. GA-BiFPN focuses on geometry-aware multi-scale fusion. Instead of relying only on appearance-based feature aggregation, it introduces coarse-depth-derived structural cues into the fusion process. The depth-gradient cue highlights potential structural transitions in the predicted scene geometry, while the gated injection mechanism allows the network to adaptively balance the original fused feature and the geometry-aware feature at each scale. Therefore, GA-BiFPN is used to strengthen structural representation in weak-texture regions and around tissue boundaries.

ISA focuses on illumination-robust bottleneck enhancement. Endoscopic illumination artifacts may cause abnormal feature responses that are not directly related to tissue geometry. The illumination branch performs global channel recalibration to suppress such responses, while the semantic branch preserves tissue-related structural patterns before depth decoding. The residual connection further allows ISA to behave as an incremental feature correction module, reducing the risk of discarding useful structural information. In this way, GA-BiFPN and ISA provide complementary feature-level adaptations: the former emphasizes geometry-aware structural fusion, whereas the latter improves illumination-robust semantic representation.

### Offline multi-generation self-distillation accuracy optimization strategy

In the baseline EndoDAC framework, the optimization of DepthNet is mainly driven by the self-supervised reprojection objective, where view synthesis is performed using the relative poses and camera intrinsics estimated by the Pose–Intrinsics Net, and the reconstruction error is minimized through photometric-consistency-related loss terms. However, in endoscopic scenes, phenomena such as specular reflections, shadows, local overexposure, and weak textures are widespread, making the photometric consistency assumption frequently violated and introducing noisy gradients and unstable convergence during training. Knowledge distillation and self-distillation have been widely used as training refinement strategies [30, 31]. In this work, we adapt offline multi-generation self-distillation to the proposed EndoDAC-based self-supervised endoscopic depth estimation pipeline. The motivation is to provide additional soft depth supervision in regions where photometric reprojection losses may be unreliable. Specifically, a three-generation setting is employed: a conventionally trained model with GA-BiFPN and ISA is first selected as a fixed teacher, and generation-by-generation distillation is then performed so that the student aligns its depth predictions with those of the teacher while preserving the original self-supervised objective. The best student of each generation is subsequently used to construct the teacher for the next generation, thereby achieving progressive refinement of the prediction results.

#### Teacher construction and offline distillation procedure

We first perform a standard self-supervised training using the complete model equipped with GA-BiFPN and ISA, and obtain the model parameters *θ(0)* with the best performance on the validation set. These parameters are fixed as the 0th-generation teacher model *T(0)*. Subsequently, a three-generation distillation training process is carried out ($g \in \{1,2,3\}$), and each generation follows the same procedure: the parameters of the teacher $T^{(g-1)}$ are fixed, while the student $S^{(g)}$ is initialized and trained, with the student adopting the same network architecture as the teacher and retaining both GA-BiFPN and ISA. After training is completed, the student parameters $\theta^{(g)}$ with the best performance in the current generation are selected and fixed as the teacher $T^{(g)}$ for the next generation.

This procedure follows an offline teacher setting, in which the teacher model remains fixed throughout the training of each generation and only provides stable soft target signals.

#### Optimization objective and distillation loss

In the *g*-th generation of distillation, the total loss of the student model is defined as:

10 $$L^{(g)} = L_{ss} + \lambda_g L_{kd}$$

where $L_{ss}$ denotes the baseline self-supervised reprojection reconstruction objective (including photometric-consistency-related terms and their robust design), $L_{kd}$ denotes the distillation term, and $\lambda_g$ represents the distillation weight for the *g*-th generation.

Distillation target: We perform distillation on the depth predictions. Let the predictions of the teacher and the student at a certain output scale be $D_t$ and $D_s$, respectively. To be consistent with the engineering implementation, a temperature parameter *τ* (set to *τ=1.0* in this paper) is introduced, and the predictions are compared after temperature scaling. The distillation loss is defined using a pixel-wise distance ($L_1$ is used in this paper):

11 $$\mathcal{L}_{kd} = \sum_l \left\| \psi(D_s^l; \tau) - \psi(D_t^l; \tau) \right\|_1$$

where *l* denotes the scale index, and $\psi(\cdot;\tau)$ represents the temperature mapping consistent with the implementation (when *τ=1.0*, it is equivalent to no additional softening). This distillation term does not replace $L_{ss}$, but instead serves as an additional soft geometric prior, enabling the student to obtain more stable learning signals during the early stage of training or in regions where photometric supervision is unreliable.

#### Three-generation distillation with decaying weights

We perform three generations of distillation in total, and progressively decrease the distillation weight across generations to balance the stability of teacher imitation and the adaptability to self-supervised signals. The specific hyperparameter settings are as follows (the temperature is set to *τ=1.0* for all generations): Generation 1 (*g=1*): teacher $T^{(0)}$, distillation weight $\lambda_1=1.0$; Generation 2 (*g=2*): teacher $T^{(1)}$, distillation weight $\lambda_2=0.7$; Generation 3 (*g=3*): teacher $T^{(2)}$, distillation weight $\lambda_3=0.4$.

This progressively weakened distillation constraint allows the training process to gradually transition from stronger teacher guidance for stable convergence to greater reliance on the self-supervised objective for refinement, thereby reducing the limitation of excessive imitation on the model’s adaptability in the later stages.

#### Inference stage

During inference, only the optimal student model $S^{(3)}$ from the third generation of distillation is used for depth prediction. The offline teacher participates only in the computation of the distillation loss during training and introduces no additional inference overhead. In our experimental setting, each complete training stage takes approximately 4 hours on a single NVIDIA RTX 4090 GPU; therefore, obtaining the third-generation student requires four sequential training stages, including the initial non-distilled teacher and three distillation generations, corresponding to approximately 16 hours in total.

### Structural fusion acceleration strategy for the inference stage

For deployment efficiency, we apply two equivalent fusion optimizations at the inference stage, motivated by low-rank adaptation and structural re-parameterization/operator fusion techniques [19, 20]: LoRA weight fusion and Conv–BN fusion. These operations are not part of the self-supervised training formulation; instead, they are used as inference-stage engineering optimizations to reduce redundant computation and memory access after training. Both operations are executed once after the model enters eval mode and preserve the prediction behavior up to negligible floating-point rounding differences. With this acceleration strategy, the inference time for a single image is reduced from 8.8 ms to 7.3 ms under the same hardware and testing settings.

#### LoRA weight fusion

During training, DV-LoRA learns incremental parameters by introducing a low-rank bypass branch alongside the frozen base weights. If used directly during inference, additional matrix operations from the bypass branch are required. Before inference, we fuse the incremental weight *Δ W* learned by the LoRA branch into the base weight $W_0$, resulting in the fused weight $W_{\mathrm{fused}} = W_0 + \Delta W$. Thereafter, inference is performed using only a single computation path, thereby eliminating the additional computational overhead introduced by LoRA. For the dynamic vector modulation form of DV-LoRA, the fusion process computes the corresponding incremental weight according to its parameterization.

#### Conv–BN fusion

We fold the affine transformation parameters of BN into the weights and bias of the preceding convolution layer during inference preparation, and replace the BN layer with an identity mapping [20], thereby reducing the number of operators and memory accesses and improving inference speed.

The above fusion operations are inference-stage equivalent transformations, introducing only negligible differences due to floating-point rounding and leaving the training process unchanged. The final deployed model performs forward prediction using the fused model. To quantitatively verify that the fusion operations do not degrade prediction accuracy, we further compare the depth estimation metrics before and after inference-time structural fusion in the Results section.

### Implementation details

SCARED dataset [32]: SCARED was originally developed for the MICCAI 2019 EndoVis challenge and contains 35 endoscopic video sequences totaling 22,950 frames. The videos were acquired using a da Vinci Xi endoscope in fresh porcine cadaver abdominal anatomical scenes. Each video sequence provides ground-truth depth maps captured by a projector, along with the corresponding ground-truth camera poses and camera intrinsic parameters. Following the data split adopted by the baseline, we divide SCARED into a training set of 15,351 frames, a validation set of 1,705 frames, and a test set of 551 frames.

Hamlyn dataset (zero-shot): Hamlyn is an endoscopic video dataset collected from various surgical procedures and contains challenging real in vivo scenes. Following the selection protocol adopted by the baseline, 21 video sequences are used for evaluation. Consistent with the baseline setting, the model trained on SCARED is directly applied to Hamlyn for zero-shot testing.

Unless otherwise specified, all experimental settings follow those in [18]. All experiments are conducted on a single NVIDIA RTX 4090 GPU. For repeated-run stability analysis, the final proposed model is trained three times using different random seeds (314, 23, and 2026), and the results are reported as mean *±* standard deviation.

Additional hyperparameters: In addition to the baseline settings, we introduce GA-BiFPN and ISA. GA-BiFPN enables geometric injection, uses depth gradients as geometric cues by default, and adopts gated fusion; the number of BiFPN stacking layers is set to 2. In terms of the training strategy, we employ offline multi-generation self-distillation with a temperature of *τ=1.0*, and set the distillation weights for the three generations to 1.0 / 0.7 / 0.4, respectively. Distillation starts from epoch 0 and is applied only to the depth outputs. During deployment, LoRA weight fusion and Conv–BN fusion are enabled to improve inference efficiency. The evaluation metrics are consistent with those of the baseline, including Abs Rel, Sq Rel, RMSE, RMSE(log), and threshold accuracy *δ*, with median scaling applied for scale alignment.

## Results

### Comparative experiments

We evaluate the proposed model against representative existing methods under two testing scenarios: in-domain testing on SCARED and zero-shot testing on Hamlyn. Quantitative results on the SCARED test set demonstrate the model’s performance on a standard benchmark. Zero-shot evaluation on Hamlyn provides an additional SCARED-to-Hamlyn transfer assessment under a different data distribution.

In-domain Evaluation on SCARED. Table 1 summarizes the quantitative comparison with several publicly available methods. The proposed EndoDAC-based extension achieves the best results among the compared methods on the major error metrics and threshold accuracy. These results suggest that the task-specific integration of GA-BiFPN, ISA, and offline multi-generation self-distillation improves the baseline pipeline on the SCARED benchmark. In particular, compared with EndoDAC, the improvements in Sq Rel and RMSE indicate that the proposed adaptations are helpful for reducing relatively large depth estimation errors.

**Table 1:** Comparison results with mainstream methods on the SCARED dataset

Method	Year	Abs Rel*↓*	Sq Rel*↓*	RMSE*↓*	RMSE log*↓*	*δ**↑*
AF-SfMLearner [3]	2022	0.059	0.435	4.925	0.082	0.974
MonoViT [33]	2022	0.062	0.470	5.042	0.082	0.976
LiteMono [14]	2023	0.057	0.453	4.967	0.079	0.975
Surgical-DINO [34]	2024	0.059	0.427	4.904	0.081	0.974
Yang et al. [35]	2024	0.062	0.558	5.585	0.090	0.962
Depth Anything [15]	2024	0.058	0.451	5.058	0.081	0.974
Depth Anything v2 [36]	2024	0.075	0.686	6.322	0.105	0.948
IID-SfMLearner [37]	2024	0.058	0.435	4.820	0.080	0.969
DVSMono [38]	2024	0.055	0.410	4.797	0.078	0.975
DARES [39]	2024	0.052	0.356	4.483	0.073	0.980
EndoDAC [18]	2024	0.052	0.362	4.464	0.073	0.979
SfM-Diffusion [40]	2025	0.049	0.366	4.305	0.078	0.975
MonoPCC [41]	2025	0.051	0.349	4.488	0.072	0.983
**Ours**	-	**0.047**	**0.307**	**4.148**	**0.067**	**0.985**

The best results are shown in bold

Zero-shot Cross-domain Evaluation on Hamlyn. Table 2 reports the results of zero-shot testing on the Hamlyn dataset, where the model is trained exclusively on SCARED and directly applied to Hamlyn without any self-supervised fine-tuning, parameter updating, or geometric re-estimation. This setting provides a limited SCARED-to-Hamlyn transfer assessment under real in vivo scenes and cross-device imaging conditions. Under this evaluation protocol, the proposed method achieves lower Abs Rel, Sq Rel, RMSE, and RMSE log than the EndoDAC baseline, while its threshold accuracy *δ* remains slightly lower. These results suggest a partial improvement in cross-domain error performance, mainly reflected in the reduction of error-oriented metrics. However, the lower *δ* value indicates that threshold-level accuracy and fine-grained scale consistency are not fully improved in the zero-shot setting.

**Table 2:** Comparison results with mainstream methods on the Hamlyn dataset in the zero-shot setting

Method	Year	Abs Rel*↓*	Sq Rel*↓*	RMSE*↓*	RMSE log*↓*	*δ**↑*
AF-SfMLearner [3]	2022	0.168	4.440	13.870	0.204	0.770
MonoViT [33]	2022	0.193	10.512	18.028	0.220	0.769
LiteMono [14]	2023	0.179	6.366	15.196	0.216	0.754
IID-SfMLearner [37]	2024	0.171	4.526	14.066	0.206	0.767
Depth Anything v2 [36]	2024	0.182	4.994	15.067	0.219	0.740
DARES [39]	2024	0.164	4.456	13.625	0.201	0.783
EndoDAC [18]	2024	0.156	3.854	12.936	0.193	**0.791**
MonoPCC [41]	2025	0.158	3.889	13.205	0.194	0.782
**Ours**	-	**0.154**	**3.562**	**12.468**	**0.188**	0.789

The best results are shown in bold

### Repeated-run stability analysis

To further assess the robustness of the proposed method, we repeated the training of the final model three times using different random seeds (314, 23, and 2026) under the same data split and evaluation protocol. The models trained on SCARED were evaluated on both the SCARED test set and the Hamlyn zero-shot test set. The results are reported as mean *±* standard deviation over three runs.

As shown in Table 3, the proposed method shows stable performance across different random seeds. On SCARED, the standard deviations are small for all metrics, indicating that the in-domain results are not caused by an unstable single training run. On Hamlyn, the zero-shot results also remain stable across the three trained models, with limited variations in Sq Rel, RMSE, and RMSE log, while Abs Rel and *δ* show variations smaller than the displayed numerical precision. These results support the robustness of the reported performance of the proposed method, although the cross-domain improvements should still be interpreted cautiously because the margin over EndoDAC is modest.

**Table 3:** Repeated-run stability analysis of the proposed method on SCARED and Hamlyn. Results are reported as mean*±*standard deviation over three runs

Dataset	Abs Rel*↓*	Sq Rel*↓*	RMSE*↓*	RMSE log*↓*	*δ**↑*
SCARED	$0.047 \pm { < }0.001$	*0.307 ± 0.015*	*4.148 ± 0.030*	*0.067 ± 0.001*	$0.985 \pm { < }0.001$
Hamlyn	$0.154 \pm { < }0.001$	*3.562 ± 0.050*	*12.468 ± 0.100*	*0.188 ± 0.001*	$0.789 \pm { < }0.001$

The final model is trained three times using random seeds 314, 23, and 2026 under the same SCARED train-ing split. Hamlyn is evaluated in the zero-shot setting using the corresponding SCARED-trained models. Standard deviations smaller than 0.001 are reported as < 0:001 due to the displayed numerical precision

### Verification of inference-time structural fusion

As shown in Table 4, the evaluation metrics remain unchanged after applying inference-time structural fusion, while the single-frame inference latency is reduced from 8.8 ms to 7.3 ms. This result verifies that LoRA weight fusion and Conv-BN fusion act as inference-time re-parameterization operations and improve deployment efficiency without degrading depth estimation accuracy.

**Table 4:** Accuracy and latency comparison before and after inference-time structural fusion on the SCARED dataset

Setting	LoRA	Conv-BN	Abs Rel*↓*	Sq Rel*↓*	RMSE*↓*	RMSE log*↓*	*δ**↑*	Latency*↓*
Before fusion	*×*	*×*	0.047	0.307	4.148	0.067	0.985	8.8 ms
After fusion	*\checkmark*	*\checkmark*	0.047	0.307	4.148	0.067	0.985	7.3 ms

Both settings are evaluated using the same Gen-3 checkpoint and the same evaluation protocol. LoRA denotes LoRA weight fusion, and Conv-BN denotes convolution-batch normalization fusion. Inference-time structural fusion is applied only in evaluation mode and does not involve retraining

### Ablation studies

For integrated medical imaging pipelines composed of multiple representation-enhancement and optimization components, component-wise evaluation is important for isolating the contribution of each design and avoiding over-interpretation of aggregate gains [42, 43]. In this section, ablation analyses are conducted on the key adaptations of the proposed EndoDAC-based extension on the SCARED dataset under the same training and evaluation protocol as the baseline. We first verify the contribution of the structural enhancement modules, GA-BiFPN and ISA, to performance. Subsequently, the generation-by-generation results of the offline multi-generation self-distillation strategy are presented to analyze the gains and trends brought by the training strategy.

As shown in Table 5, introducing GA-BiFPN alone reduces Abs Rel from 0.052 to 0.049 and RMSE from 4.464 to 4.403, indicating that geometry-aware bidirectional feature fusion improves multi-scale representation and structural consistency. Introducing ISA alone also yields performance gains, reducing RMSE from 4.464 to 4.402, which suggests that illumination-semantic feature refinement helps reorganize bottleneck features under adverse illumination conditions in endoscopic scenes.

**Table 5:** Ablation results of GA-BiFPN and ISA with parameter-matched controls on the SCARED dataset

Setting	BiFPN	Geo.	ISA	Params	Abs Rel*↓*	Sq Rel*↓*	RMSE*↓*	RMSE log*↓*	*δ**↑*
Baseline	*×*	*×*	*×*	1.67 M	0.052	0.362	4.464	0.073	0.979
+ Plain BiFPN	*\checkmark*	*×*	*×*	9.75 M	0.052	0.373	4.622	0.074	0.979
+ GA-BiFPN	*\checkmark*	*\checkmark*	*×*	9.85 M	0.049	0.340	4.403	0.070	**0.983**
+ ISA	*×*	*×*	*\checkmark*	2.50 M	0.051	0.359	4.402	0.073	0.981
+ Plain BiFPN + ISA	*\checkmark*	*×*	*\checkmark*	10.59 M	0.050	0.371	4.606	0.072	0.980
+ GA-BiFPN + ISA	*\checkmark*	*\checkmark*	*\checkmark*	10.69 M	**0.048**	**0.339**	**4.395**	**0.069**	**0.983**

Plain BiFPN denotes the BiFPN-based multi-scale fusion module without coarse-depth-derived geometry injection. Geo. denotes the proposed depth-gradient-guided gated geometry injection. Params indicates the number of trainable parameters of the DepthNet. All models in this table are trained without knowledge distillation. The best or tied-best results are shown in bold

To provide a fairer comparison under a similar parameter scale, we further include Plain BiFPN variants as capacity-matched controls. Plain BiFPN retains the BiFPN-style multi-scale fusion structure but removes the coarse-depth-derived geometry injection. Compared with GA-BiFPN, Plain BiFPN has a comparable number of trainable parameters, but it does not improve the baseline in this setting, with Sq Rel and RMSE increasing to 0.373 and 4.622, respectively. In contrast, GA-BiFPN achieves lower errors under a similar parameter scale, suggesting that the geometry-aware injection mechanism contributes to more effective multi-scale structural modeling.

A consistent trend is observed when ISA is enabled. Plain BiFPN + ISA obtains an Abs Rel of 0.050 and an RMSE of 4.606, whereas GA-BiFPN + ISA further improves these metrics to 0.048 and 4.395, respectively. Since these two variants have comparable trainable parameter counts, the comparison indicates that the improvement is associated not only with increased model capacity, but also with the proposed geometry-aware fusion design. Overall, GA-BiFPN and ISA achieve the best performance when used together, demonstrating the complementarity between geometry-aware multi-scale fusion and illumination-semantic feature refinement.

As shown in Table 6, offline multi-generation self-distillation yields stable improvements that gradually saturate on the SCARED test set. Compared with the model without distillation, the first generation improves the major metrics overall. Abs Rel reduces from 0.048 to 0.047, Sq Rel from 0.339 to 0.315, RMSE from 4.395 to 4.217, and RMSE(log) from 0.069 to 0.068. Meanwhile *δ* increases from 0.983 to 0.984. In the second and third generations of distillation, Abs Rel remains at 0.047, indicating a performance plateau, whereas Sq Rel and RMSE continue to decrease (0.315*→*0.310*→*0.307 and 4.217*→*4.177*→*4.148, respectively), RMSE(log) is further reduced to 0.067, and *δ* increases to 0.985 and remains stable. These results indicate that the gains of multi-generation distillation are mainly reflected in the further suppression of mismatches in samples with relatively large errors, as evidenced by the continuous reduction in Sq Rel and RMSE. In contrast, the overall relative error, represented by Abs Rel, is already close to saturation after the first generation, and therefore exhibits only limited subsequent improvement. Considering the additional offline training cost, the third-generation model is used in this paper as an accuracy-oriented setting, while the first or second generation may provide a more cost-effective choice when training resources are limited. Based on the final accuracy-oriented setting, the model obtained from the third generation of distillation is used in all subsequent comparative experiments.

**Table 6:** Generation-by-generation comparison of offline multi-generation self-distillation on the SCARED dataset

Setting	Abs Rel*↓*	Sq Rel*↓*	RMSE*↓*	RMSE log*↓*	*δ**↑*
No KD	0.048	0.339	4.395	0.069	0.983
Gen-1 (*λ=1.0*)	**0.047**	0.315	4.217	0.068	0.984
Gen-2 (*λ=0.7*)	**0.047**	0.310	4.177	**0.067**	**0.985**
Gen-3 (*λ=0.4*)	**0.047**	**0.307**	**4.148**	**0.067**	**0.985**

"No KD" indicates training without knowledge distillation.  λ denotes the weight of the distillation loss term. The best or tied-best results are shown in bold

### Visualization results

**Fig. 4:**
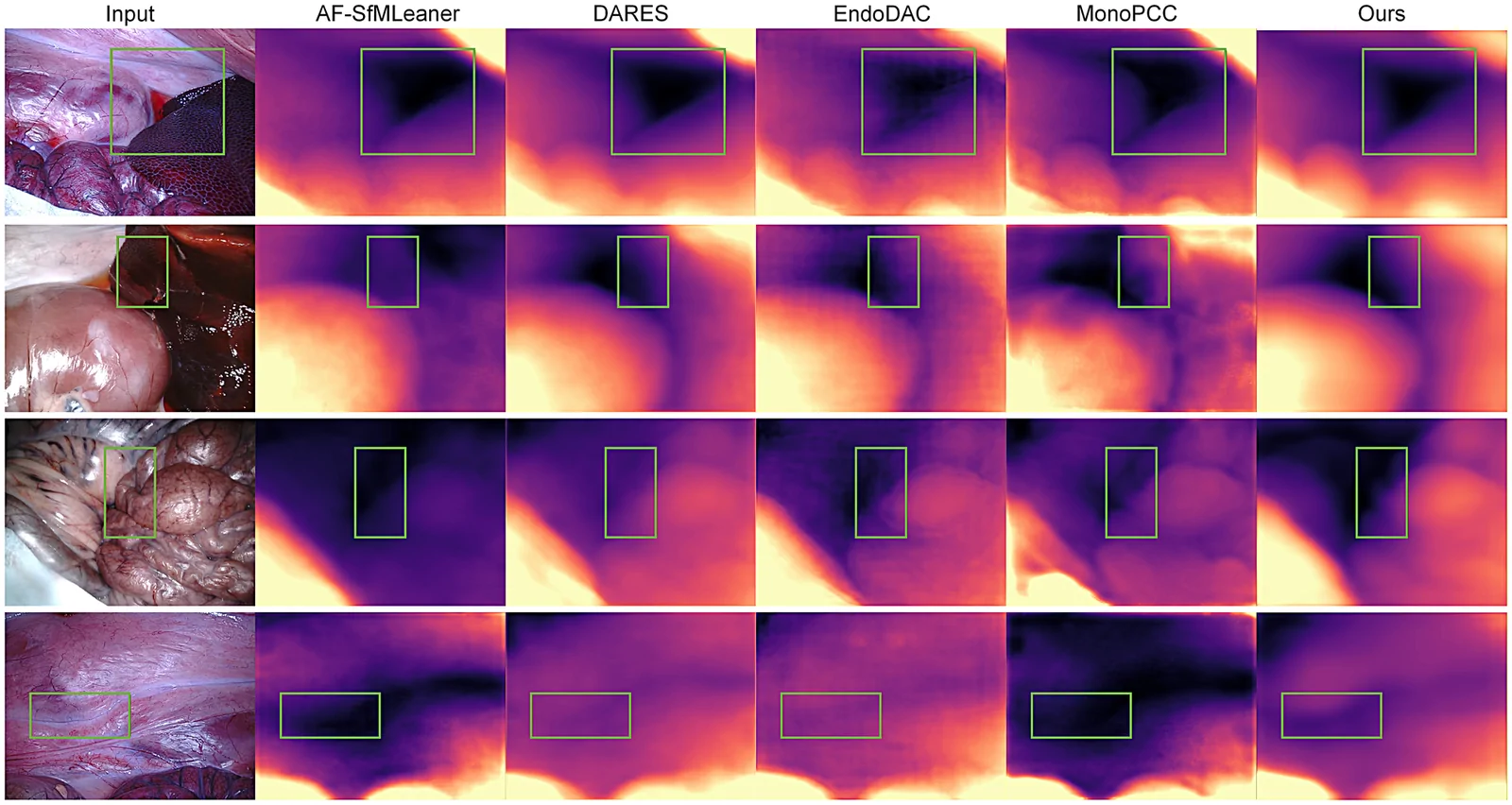
Qualitative results of depth estimation on the SCARED dataset

As shown in Fig. 4, we present a comparative visualization of the disparity prediction results produced by five methods on representative endoscopic frames. Overall, there are clear differences among the methods in terms of spatial continuity and structural consistency around tissue boundaries, weak-texture regions, and specular reflection areas. From visual inspection, methods with weaker performance tend to exhibit abnormal disparity voids in specular highlight regions, or suffer from structure collapse caused by excessive smoothing in weak-texture flat regions. They are also prone to blurred boundaries and local discontinuities in high-frequency regions such as vessel edges. In contrast, the disparity map produced by the proposed method demonstrates better spatial continuity and boundary consistency in these challenging regions: it more effectively suppresses local abnormal responses around specular highlights, preserves reasonable depth gradient variations in weak-texture areas, and presents clearer hierarchical transitions at tissue structural boundaries. This phenomenon is consistent with the design motivation of the proposed method: GA-BiFPN enhances structural constraints by guiding multi-scale fusion with geometric cues, while ISA suppresses illumination disturbances and strengthens semantic structural representation before decoding, thus enhancing spatial continuity and structural consistency under non-ideal imaging conditions.

**Fig. 5:**
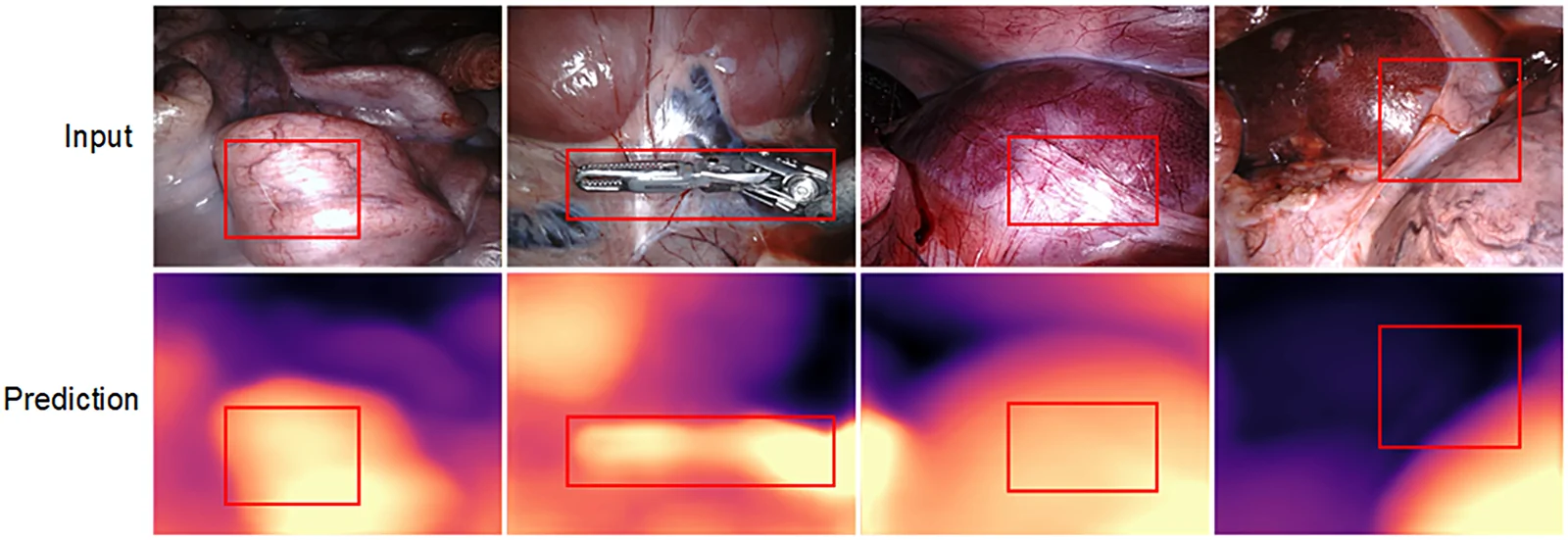
Representative failure cases of the proposed method. Each column shows an endoscopic input image in the top row and its corresponding predicted depth map in the bottom row.

As shown in Fig. 5, the proposed method still exhibits limitations under challenging imaging conditions. Several failure cases are associated with severe specular reflection, local overexposure, weak tissue texture, and surgical instrument occlusion. In these regions, the predicted depth maps tend to show locally unstable responses, over-smoothed transitions, or reduced boundary consistency. Since these factors weaken the reliability of photometric cues and obscure tissue structures, they remain challenging for self-supervised monocular endoscopic depth estimation. These examples indicate that, although the proposed method improves overall performance, robust prediction under extreme illumination, occlusion, and severe texture ambiguity still requires further improvement.

## Discussion

The experiments mainly validate three aspects of the proposed EndoDAC-based adaptation: feature enhancement, training refinement, and inference-stage efficiency. The ablation results on SCARED show that GA-BiFPN and ISA each improve the baseline, and their combination achieves the best performance among the tested module configurations. This suggests that geometry-aware multi-scale fusion and illumination-semantic bottleneck enhancement provide complementary benefits within the same self-supervised pipeline. The improvements in Sq Rel and RMSE further indicate that the proposed feature adaptations are helpful for reducing relatively large local depth errors. The repeated-run analysis further shows that the proposed method maintains stable performance across different random seeds on both SCARED and Hamlyn, although the cross-domain margin over EndoDAC remains modest.

The generation-by-generation distillation results show that offline self-distillation improves accuracy, but the gains gradually saturate. Most of the improvement appears after the first distillation generation, while later generations mainly bring smaller reductions in Sq Rel, RMSE, and RMSE log. In our experimental setting, obtaining the third-generation student requires approximately 16 hours of sequential training on a single NVIDIA RTX 4090 GPU. Therefore, the third-generation model is used as an accuracy-oriented setting, whereas fewer distillation generations may be more practical when training resources are limited. Since only the final student model is used during inference, this training cost does not introduce additional runtime overhead.

The Hamlyn zero-shot evaluation provides a limited SCARED-to-Hamlyn transfer assessment. The proposed method improves several error-oriented metrics over EndoDAC, but the threshold accuracy *δ* remains comparable to, rather than clearly better than, the baseline. Therefore, the results should be interpreted as partial improvement in cross-domain error performance rather than comprehensive evidence of cross-domain robustness. This also indicates that fine-grained scale consistency under domain shift remains an unresolved issue.

Several limitations should be noted. First, the current evaluation is based mainly on the SCARED-to-Hamlyn transfer setting, and further validation on more diverse endoscopic datasets, imaging conditions, and backbone architectures is required. Second, the method still needs to be tested under more challenging cases, such as severe occlusion, strong deformation, and extreme illumination variation. Third, although multi-generation self-distillation improves accuracy, it increases offline training cost. Future work will focus on improving cross-domain scale consistency, reducing training complexity, and enhancing robustness under more diverse surgical conditions.

## Conclusions

This paper addresses the limitations of self-supervised monocular depth estimation in challenging endoscopic scenes. Building upon the EndoDAC framework, we presented a task-specific architectural extension that integrates geometry- and illumination-aware feature enhancement, offline multi-generation self-distillation, and inference-stage structural fusion. The proposed method preserves the original self-supervised photometric reprojection formulation, while improving feature representation, training refinement, and deployment efficiency for endoscopic scenarios.

Experimental results on SCARED show that the proposed EndoDAC-based extension achieves consistent improvements over the baseline and several compared methods. Zero-shot evaluation on Hamlyn further shows improved error-oriented metrics compared with EndoDAC, although threshold accuracy and cross-domain scale consistency remain limited. These findings indicate that task-specific architectural adaptation can improve self-supervised endoscopic depth estimation under the evaluated setting, but also suggest that broader cross-domain robustness remains insufficiently validated and that domain shift in real in vivo scenes remains an important challenge. Future work will focus on robustness under extreme illumination, heavy occlusion, and significant deformation, and will explore geometric constraints that rely less on photometric consistency, more effective cross-domain adaptation, and more efficient training strategies.

## Data Availability

The datasets analysed during the current study were obtained from their original dataset sources. The SCARED dataset (Stereo Correspondence and Reconstruction of Endoscopic Data) was originally released as part of the MICCAI 2019 Endoscopic Vision Challenge / EndoVis SCARED sub-challenge [32]. Dataset information and access instructions are available through the Grand Challenge platform at https://endovissub2019-scared.grand-challenge.org/. Access to the SCARED data is subject to the dataset providers’ access procedure, including contacting the providers and completing the required data-use agreement. The Hamlyn data used in this study were obtained from the rectified Hamlyn dataset with ground-truth depth released by the Endo-Depth-and-Motion project [44]. The project page is available at https://davidrecasens.github.io/EndoDepthAndMotion/. This resource provides rectified stereo images, calibration files, ground-truth depth maps, trained Endo-Depth models, and data splits for the Hamlyn Centre laparoscopic in vivo endoscopy stereo video dataset. The ground-truth depth maps were generated using the stereo matching software Libelas, as described by the dataset providers.

## Code availability

The code used in this study is publicly available at https://github.com/Bq1207/MonoGID.

## List of abbreviations

3D: Three-dimensional
Abs Rel: Absolute Relative Error
BiFPN: Bidirectional Feature Pyramid Network
BN: Batch Normalization
Conv: Convolution
GA-BiFPN: Geometry-Aware Bidirectional Feature Pyramid Network
GT: Ground Truth
ISA: Illumination-Semantic Attention
KD: Knowledge Distillation
LoRA: Low-Rank Adaptation
MICCAI: Medical Image Computing and Computer Assisted Intervention
ORB-SLAM: Oriented FAST and Rotated BRIEF Simultaneous Localization and Mapping
RMSE: Root Mean Square Error
SCARED: Stereo Correspondence and Reconstruction of Endoscopic Data
SE: Squeeze-and-Excitation
Sq Rel: Squared Relative Error
